# TAK1 Reduces Surgery-induced Overactivation of RIPK1 to Relieve Neuroinflammation and Cognitive Dysfunction in Aged Rats

**DOI:** 10.1007/s11064-023-03959-z

**Published:** 2023-06-17

**Authors:** Yuhan Zhang, Yang Su, Ziheng Wang, Teng Li, Liwei Wang, Daqing Ma, Meiyan Zhou

**Affiliations:** 1 Department of Anesthesiology, Xuzhou Central Hospital, Xuzhou, 221009 China; 2 Jiangsu Province Key Laboratory of Anesthesiology, Xuzhou Medical University, Xuzhou, 221004 China; 3 Division of Anaesthetics, Pain Medicine and Intensive Care, Department of Surgery and Cancer, Faculty of Medicine, Imperial College London, Chelsea and Westminster Hospital, London, UK

**Keywords:** Postoperative cognitive dysfunction, Neuroinflammation, Receptor-interacting protein kinase 1, Transforming growth factor β-activated kinase 1, Ageing

## Abstract

Background: Postoperative cognitive dysfunction (POCD) is a common clinical complication in elderly patients, but its underlying mechanism remains unclear. Receptor-interacting protein kinase 1 (RIPK1), a key molecule mediating necroptosis and regulated by transforming growth factor β-activated kinase 1 (TAK1), was reported to be associated with cognitive impairment in several neurodegenerative diseases. This study was conducted to investigate the possible role of TAK1/RIPK1 signalling in POCD development following surgery in rats. Methods: Young (2-month-old) and old (24-month-old) Sprague–Dawley rats were subjected to splenectomy under isoflurane anaesthesia. The young rats were treated with the TAK1 inhibitor takinib or the RIPK1 inhibitor necrostatin-1 (Nec-1) before surgery, and old rats received adeno-associated virus (AAV)-TAK1 before surgery. The open field test and contextual fear conditioning test were conducted on postoperative day 3. The changes in TNF-α, pro-IL-1β, AP-1, NF-κB p65, pRIPK1, pTAK1 and TAK1 expression and astrocyte and microglia activation in the hippocampus were assessed. Results: Old rats had low TAK1 expression and were more susceptible to surgery-induced POCD and neuroinflammation than young rats. TAK1 inhibition exacerbated surgery-induced pRIPK1 expression, neuroinflammation and cognitive dysfunction in young rats, and this effect was reversed by a RIPK1 inhibitor. Conversely, genetic TAK1 overexpression attenuated surgery-induced pRIPK1 expression, neuroinflammation and cognitive dysfunction in old rats. Conclusion: Ageing-related decreases in TAK1 expression may contribute to surgery-induced RIPK1 overactivation, resulting in neuroinflammation and cognitive impairment in old rats.

## Introduction

Ageing is considered to be a chronic low-grade proinflammatory condition that is a strong risk factor for frailty, multimorbidity, and physical and cognitive disability [1, 2]. Postoperative cognitive dysfunction (POCD) is more common in elderly patients, and it prolongs hospitalization, reduces quality of life, increases postoperative morbidity and mortality, and imposes a serious burden for individuals and society[3–5]. The crucial role of neuroinflammation in the pathophysiological mechanism underlying POCD has been well documented [6, 7]. However, it remains to be elucidated how neuroinflammation is initiated in the aged brain after surgery.

Receptor-interacting protein kinase (RIPK1) mediates necroptotic cell death, induces ischaemic organ injury [8, 9], and plays a role in chronic neurodegenerative conditions, such as multiple sclerosis [10], amyotrophic lateral sclerosis [11] and Alzheimer’s disease [12–14]. A recent study showed that inhibiting RIPK1 activation suppressed neuroinflammation and attenuated cognitive impairment following surgery[15]. However, the underlying mechanisms by which RIPK1 is activated following surgery remain unknown.

Transforming growth factor β-activated kinase 1 (TAK1) is an endogenous RIPK1 inhibitor that can directly inhibit RIPK1 activation through phosphorylation to promote RIPK1-dependent apoptosis [16–18]. The expression of TAK1 is decreased in elderly patients. Previous studies showed that an age-dependent reduction in TAK1 expression is associated with neurodegenerative diseases, such as frontotemporal dementia/amyotrophic lateral sclerosis, and inhibition of RIPK1 was found to alleviate disease-like symptoms[18]. It is well known that age is also a strong risk factor for POCD development[19, 20]. Whether an age-dependent decrease in TAK1 expression enhances RIPK1 activity, thereby contributing to POCD development after surgery, remains unknown. Accordingly, we investigated the possible role of TAK1 and pRIPK1 and their association in the development of surgery-induced POCD in young and old rats.

## Materials and methods

### Animals

Healthy male Sprague–Dawley rats (2-month-old young rats weighing 200–250 g and 24-month-old aged rats weighing 550–650 g) were provided by the Animal Centre of Xuzhou Medical University. They were kept under standard conditions on a 12-hour light/dark cycle at a temperature of 22–25 °C and humidity of 40–60% and provided free access to food and water. All animals were allowed to acclimate to the environment for two weeks before the experiments were carried out. This experiment was approved by the Experimental Animal Ethics Committee of Xuzhou Medical University, Jiangsu, China, and all experimental procedures were performed in accordance with the relevant guidelines of the International Pain Research Society and the Code for the Use of Laboratory Animals.

### Experimental Groups

This experiment was divided into three parts. In the first part, young and old rats were randomly divided into the control group (C) and surgery group (S). In the second part, young rats were randomly divided into the following groups: the dimethyl sulfoxide (DMSO) group, the takinib (TAK1 inhibitor; 10 µM; S8663; Selleck, China) group, and the takinib + necrostatin-1 (Nec-1; RIPK1 inhibitor; 50 µM; S8037; Selleck, China) group; the rats in these groups were administered the appropriate drugs via lateral cerebral ventricle injection 30 min before surgery. In the third part, old rats were randomly divided into the following 2 groups: the AAV-VEH group and the AAV-TAK1 group; the rats in these groups received injection of adeno-associated virus (AAV) with or without TAK1 vector into the CA1 region 21 days before surgery.

### Stereotaxic Injection

Twenty-one days before surgery, old rats were anaesthetized by intraperitoneal injection of sodium pentobarbital (40 mg/kg body weight) and secured on a stereotaxic apparatus. After holes were made bilaterally in the skull at coordinates selected according to a rat brain atlas (-3.6 mm AP, ± 1.80 mm ML, and − 3.00 mm DV)[21], hSyn promoter-Map3k7-EGFP-3FLAG-SV40 PolyA vector (AAV-TAK1) or control hSyn promoter-MCS-EGFP-3FLAG-SV40 PolyA vehicle (AAV-VEH) (GeneChem, Shanghai, China) (titer > 1.0 × 10^12^) was bilaterally injected into the CA1 region of the hippocampus (1.5 µL/side). After injection, the syringe was left in place for another 10 min. For the inhibitor treatments, 30 min before surgery, young rats were stereotaxically injected bilaterally into the lateral cerebral ventricle with takinib (10 µM), takinib + Nec-1 (50 µM) or an equal concentration of DMSO (5 µL; Sigma–Aldrich Co., St. Louis, MO, USA) through holes in the skull (-0.80 mm AP, ± 1.50 mm ML, and − 4.00 mm DV)[21] made under anaesthesia as described above for the old rats. After injections, the incision was closed, and the rats were placed on a heated blanket for recovery before surgery.

### Surgical Procedures

Splenectomy was performed under isoflurane anaesthesia as described previously[22]. After the treatments described above, the rats in the surgery group were anaesthetized, and anaesthesia was maintained with 3% and 1.5% isoflurane. An incision was made 1.5–2.0 cm below the costal margin, and the spleen was mobilized, isolated, and excised. The wound was infiltrated with 0.25% bupivacaine before the abdominal cavity was closed for postoperative pain relief. After recovering from anaesthesia, the rats were returned to cages and housed individually.

### Open Field test

We used the open field test (OFT) to evaluate the locomotor activity of the rats used for the different parts of the experiment. The rats were allowed to move freely in an open field arena (100 × 100 × 40 cm) for 5 min, and then the movement of each rat was tracked and recorded with the ANY-maze software system. The total distance travelled was used to evaluate locomotor activity.

### Contextual fear Conditioning

Contextual fear conditioning (CFC) tests were performed as described in previous studies[23, 24]. The rats underwent fear conditioning training before surgery. First, the rats were placed into the conditioning chamber and allowed to explore freely for 5 min. Then, a sound stimulus (2.2 kHz, 96 dB, 30 s) was given, and a single shock (2.0 mA, 2 s) was delivered during the last 2 s of the sound stimulus. After the shock, the rats were maintained in the chamber for 30 s and were then returned to their home cages. The context test was performed 72 h after training to test hippocampus-dependent memory. In the context test, the rats were placed into the same chamber and kept there for 180 s without exposure to shocks or tones. The time the rats spent freezing during this period was recorded and analysed with computer software (Med Associates, Inc., USA).

### Western blot Analysis

After the fear conditioning tests described above, the rats were anaesthetized with sodium pentobarbital (100 mg/kg body weight, intraperitoneal injection) and immediately decapitated. The hippocampus was isolated on ice and frozen in liquid nitrogen before being stored at − 80 °C for further analysis. The samples were homogenized and centrifuged. Then, the supernatants were collected, and the protein concentration was determined by a bicinchoninic acid (BCA) protein assay kit (P0010; Beyotime, Shanghai, China). The proteins were separated on 10% gradient sodium dodecyl sulfate polyacrylamide electrophoresis (SDS–PAGE) gels and transferred to PVDF membranes (ISEQ00010; Merck Millipore, USA). The PVDF membranes were blocked in 5% nonfat milk with slow shaking for 2 h at room temperature and then incubated with primary antibodies at 4 °C overnight, including mouse anti-TAK1 (1:100; sc-166,562; Santa Cruz Biotechnology, China), rabbit anti-pTAK1(1:1000; AF3019; Affinity Biosciences, China), rabbit anti-pRIPK1 (1:1000; 53,286 S; Cell Signaling Technology, USA), rabbit anti-RIPK1 (1:1000; 17519-1-AP; PTG, China), rabbit anti-NF-κB p65 (1:500; 10745-1-AP; PTG, China), rabbit anti-AP-1 (activator protein 1) (1:1000; 24909-1-AP; PTG, China), rabbit anti-TNF-α (1:500; A11534; ABclonal, China), rabbit anti-IL-1β (1:500; A1112; ABclonal, China), and mouse anti-β-actin (1:2000; AC004; ABclonal, China). The membranes were incubated with corresponding secondary antibodies for 1 h at room temperature. A hypersensitive or extremely hypersensitive ECL detection kit (Beyotime, China) was used for development, and the densities of the protein bands were analysed by ImageJ software.

### Immunofluorescence

After the fear conditioning test described above, some rats were anaesthetized by intraperitoneal injection of sodium pentobarbital (100 mg/kg body weight), followed by intracardial perfusion of 200 ml 0.9% saline and 300 ml 4% paraformaldehyde (PFA) in 0.1 M phosphate buffer (pH 7.4). After decapitation, the brains were removed and fixed in 4% PFA for 6 h. After fixation, the brains were dehydrated in 30% sucrose solution and allowed to sink to the bottom. Brain tissue containing the hippocampus was cut, embedded, frozen, and then sectioned (30 μm thick) with a freezing microtome. The tissue sections were blocked in PBS-T containing 10% donkey serum for 2 h at room temperature and then incubated with primary antibodies at 4 °C overnight, including mouse anti-TAK1 (1:100; sc-166,562; Santa Cruz Biotechnology, China), rabbit anti-RIPK1 (1:500; 17519-1-AP; PTG, China), rabbit anti-ionized calcium binding adapter molecule 1 (Iba1) (1:100; ab178847; Abcam) and mouse anti-glial fibrillary acidic protein (GFAP) (1:300; 3670 S; Cell Signaling Technology) monoclonal antibodies. After washing with PBS three times for 5 min, the sections were incubated with corresponding secondary antibodies, including donkey anti-rabbit IgG conjugated to Alexa Fluor® 488 and donkey anti-mouse IgG conjugated to Alexa Fluor® 594 (1:500; Life Technologies; Carlsbad, CA, USA), in the dark for 2 h at 37 °C. The fluorescence intensity in the hippocampal regions was assessed under a confocal microscope (FV1000; Olympus Corp., Tokyo, Japan).

### Statistical Analysis

The data are presented as the mean ± SD; differences between two groups were analysed with unpaired *t* test, and differences among more than three groups were analysed with one-way ANOVA followed by Tukey’s *post hoc* multiple comparison test. All the data were analysed using GraphPad Prism (version 7.0; GraphPad Software, La Jolla, CA, USA) and SPSS (version 22.0; IBM SPSS Statistics, Armonk, NY, USA). A *P* value less than 0.05 was considered to be statistically significant.

## Results

### Old Rats were more Susceptible to Surgery-induced POCD and Neuroinflammation than Young Rats

**Fig. 1:**
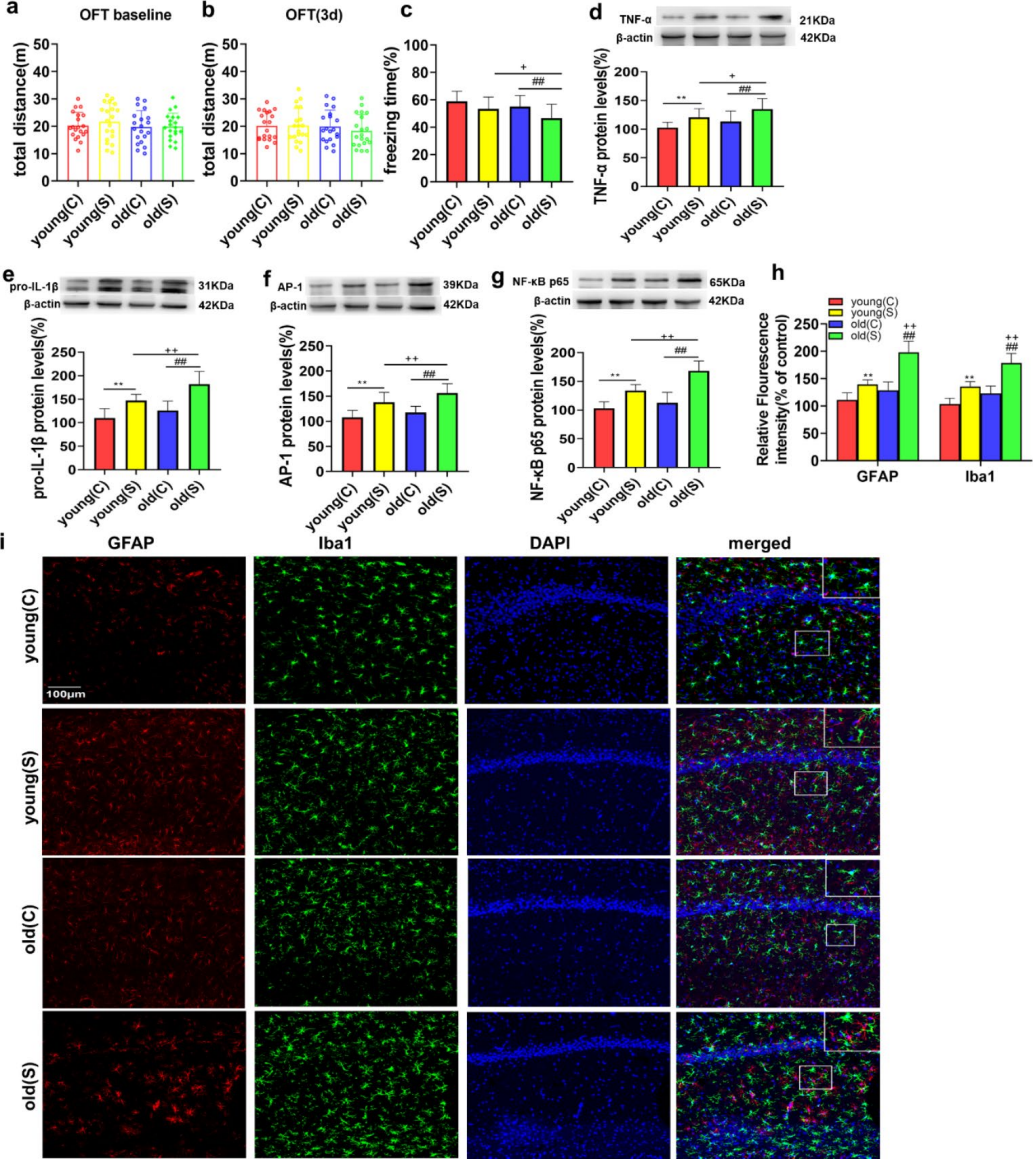
Old rats were more susceptible to surgery-induced POCD development and neuroinflammation than young rats

To evaluate how age influences the development of POCD in rats, we first assessed the locomotor activity of rats by the OFT. No significant differences in total distance travelled were detected among groups postoperatively or at baseline (Fig. 1a-b). Then, we evaluated the cognitive function of control naïve young (C) and naïve old (C) rats with CFC tests. We found that the freezing time was not significantly different between the young (C) and old (C) groups at baseline (Fig. 1c). Additionally, there was no difference in freezing time between the young rats in the control (C) and surgery (S) groups (Fig. 1c). However, the freezing time of the old surgery (S) group was significantly increased compared with that of the old control (C) and young surgery (S) groups (Fig. 1c), indicating that old rats were more susceptible to surgery-induced hippocampus-dependent cognitive decline than young rats.

No significant difference was observed in the production of proinflammatory cytokines between the naïve young (C) and old (C) groups (Fig. 1d-g). However, the levels of TNF-α, pro-IL-1β, AP-1 and NF-κB p65 in the hippocampus were increased in the young and old rats after surgery (Fig. 1d-g). Similarly, the immunofluorescence data showed that there was no difference in astrocyte and microglial activation between young (C) and old (C) rats that were not subjected to surgery (Fig. 1h-i). Surgery induced astrocyte and microglial activation in the young and old rats (Fig. 1h-i). Compared to the young (S) group, surgery-induced hippocampal neuroinflammation and astrocyte and microglial activation were markedly increased in the old (S) group (TNF-α, *P* < 0.05; pro-IL-1β, *P* < 0.01; AP-1, *P* < 0.01; NF-κB p65, *P* < 0.01; GFAP, *P* < 0.01; Iba1, *P* < 0.01; Fig. 1d-i). These data indicated that old rats were more susceptible to surgery-induced hippocampal neuroinflammation and astrocyte and microglia activation than young rats.

**(a-b)** Comparison of the total distance travelled by the rats in the OFT at different time points. **(c**) The percentage of freezing time in the 4 groups of rats in the conditioning fear contextual (CFC) test. (**d-g**) Representative western blots and analysis of TNF-α, pro-IL-1β, AP-1 and NF-κB p65 expression in hippocampal samples from the 4 groups. (**h**) Quantification of GFAP and Iba1 fluorescence in the hippocampal CA1 region in the 4 groups. (**i**) Representative images of GFAP (red) and Iba-1 (green) (markers of astrocytes and microglia, respectively) staining in the hippocampal CA1 region in the 4 groups. The data are presented as the mean ± SD (n = 20). ^**^P < 0.01 vs. the young (C) group; ^##^*P* < 0.01 vs. the old (C) group; ^+^*P* < 0.05, ^++^*P* < 0.01 vs. the young (S) group. The scale bars indicate 100 μm.

### Low TAK1 Expression, Markedly pRIPK1 in Old but not in Young Rats

**Fig. 2:**
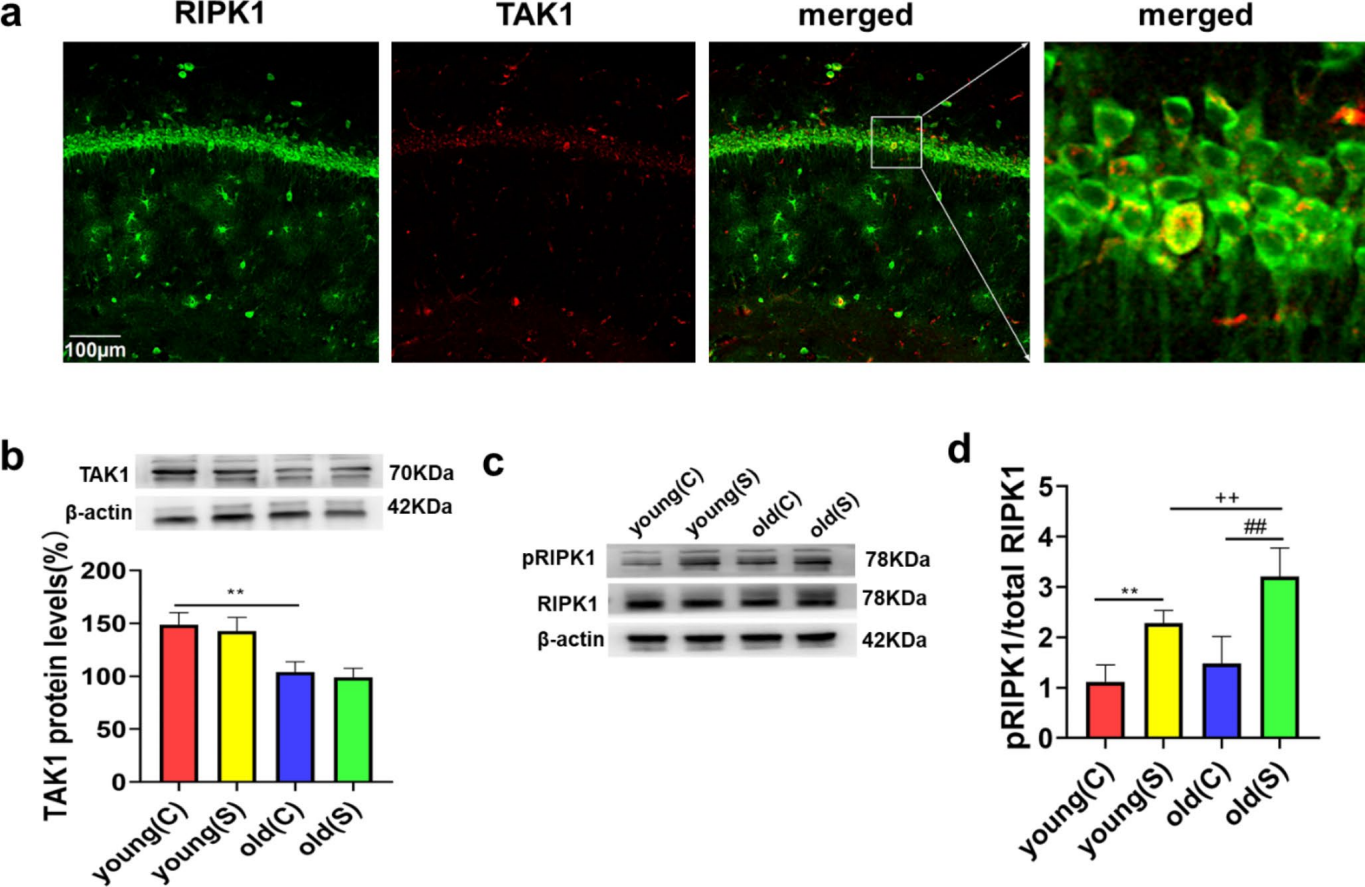
Low TAK1 expression, markedly pRIPK1 in old but not in young rats. (**a**) Double immunofluorescence staining showed that RIPK1 (green) mainly colocalized (merged) with TAK1 (red) in the hippocampal CA1 region of young rats before surgery. (**b-d**) Representative western blots and analysis of TAK1, pRIPK1, and RIPK1 expression in hippocampal samples from the 4 groups. The data are presented as the mean ± SD (n = 20). ^**^P < 0.01 vs. the young (C) group; ^##^*P* < 0.01 vs. the old (C) group; ^++^*P* < 0.01 vs. the young (S) group

The above findings suggested that age plays an important role in the development of POCD and neuroinflammation in rats, and we continued to explore the underlying mechanisms. We measured the levels of TAK1, RIPK1 and pRIPK1 in young and old rats. We found that the levels of TAK1 in the old (C) group were lower than those in the young (C) group (Fig. 2b). However, TAK1 expression was not different between the young and old rats after surgery (Fig. 2b). Surgery induced RIPK1 activation in the young and old rats (Fig. 2c-d). Compared to the young (S) group, surgery-induced RIPK1 activation was markedly increased in the old (S) group (Fig. 2c-d). However, there was no difference in the level of pRIPK1 between the young (C) and old (C) rats (Fig. 2c-d). We also found that RIPK1 mainly colocalized with TAK1 in the CA1 region of the hippocampus in young rats under physiological conditions (Fig. 2a). TAK1 and RIPK1 can be expressed in the same neuron under physiological conditions, so as to further study their interaction.

### TAK1 Inhibition Exacerbated Neuroinflammation and Cognitive Dysfunction in Young Rats After Surgery, which were Reversed by a RIPK1 Inhibitor

To determine whether TAK1 deficiency affects pRIPK1 expression, neuroinflammation and cognitive dysfunction after surgery, we first injected takinib (TAK1 inhibitor), takinib + Nec-1 (RIPK1 inhibitor) or vehicle DMSO into the CA1 region of young rats before surgery. To exclude the effect of age on TAK1 expression, young rats were used in this part of the experiment.

**Fig. 3:**
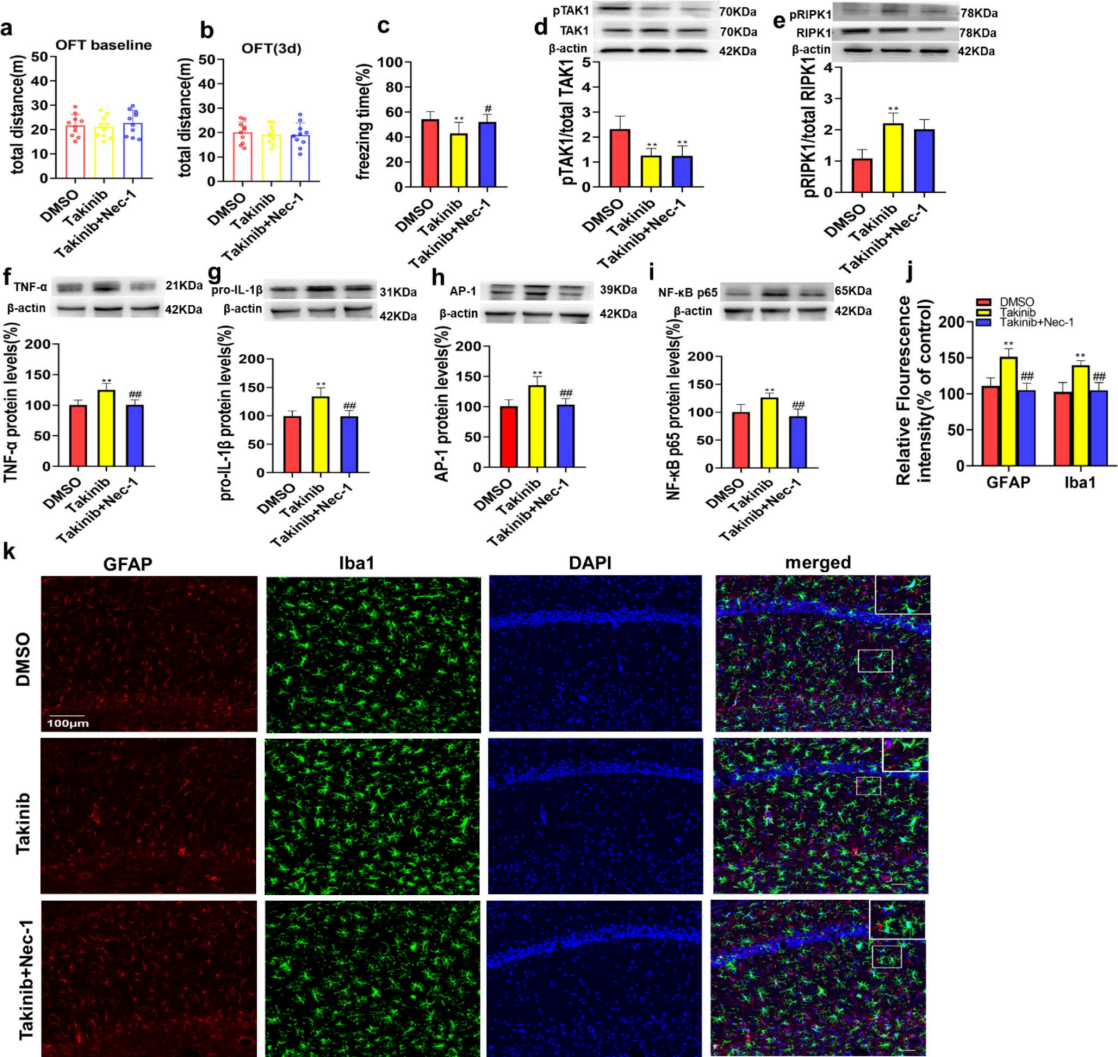
TAK1 inhibition exacerbated neuroinflammation and cognitive dysfunction in young rats after surgery, which were reversed by a RIPK1 inhibitor. **(a-b)** Comparison of the total distance travelled by the rats in the OFT at different time points. (**c)** The percentage of freezing time at 72 h after surgery. (**d-e**) Representative western blots and analysis of TAK1, pTAK1, RIPK1 and pRIPK1 expression at 72 h after surgery. (**f-i**) Representative western blots and analysis of TNF-α, pro-IL-1β, AP-1 and NF-κB p65 expression in hippocampal samples 72 h after surgery. (**j**) Quantification of GFAP and Iba1 fluorescence in the hippocampal CA1 region 72 h after surgery. (**k**) Representative images of GFAP and Iba1 fluorescence in the hippocampal CA1 region 72 h after surgery. The data are presented as the mean ± SD (n = 10). ^*^P < 0.05, ^**^P < 0.01 vs. the DMSO group; ^#^*P* < 0.05, ^##^*P* < 0.01 vs. the takinib group. The scale bars indicate 100 μm

In the OFT, there was no significant difference in the total distance travelled among groups during the preoperative or postoperative period (Fig. 3a-b). Then, we conducted CFC tests to evaluate the cognitive function of the 3 groups after surgery. The freezing time in the takinib group was significantly decreased compared with that in the DMSO group and takinib + Nec-1 group (Fig. 3c). However, the freezing time in the takinib + Nec-1 group was not significantly different from that in the DMSO group (Fig. 3c). These data indicated that the inhibitor did not impair the locomotor ability of the rats and that the TAK1 inhibitor exacerbated the postoperative cognitive decline in young rats, but this effect was reversed by Nec-1.

Furthermore, we measured the levels of proinflammatory cytokines and glial activation in the 3 groups 72 h after surgery by both western blotting and immunofluorescence staining. First, we tested the level of pTAK1 and found that takinib significantly inhibited the activity of TAK1(Fig. 3d). Then, we compared the levels of RIPK1 and pRIPK1 in the 3 groups. We found that the RIPK1 expression level was significantly decreased in the takinib + Nec-1 group compared with the control group and takinib group. The level of pRIPK1 was increased in the takinib group compared with the control group and takinib + Nec-1 group (Fig. 3e). The TNF-α, pro-IL-1β, AP-1 and NF-κB p65 levels in the takinib group were increased compared with those in the DMSO group and takinib + Nec-1 group (Fig. 3f-i), while the levels of inflammatory cytokines were not significantly different between the takinib + Nec-1 group and DMSO group (Fig. 3f-i). Similarly, the fluorescence intensity of Iba1 and GFAP was significantly increased in the takinib group compared with the DMSO group and takinib + Nec-1 groups (Fig. 3j-k), while the fluorescence intensity of Iba1 and GFAP was not significantly different between the takinib + Nec-1 and DMSO groups (Fig. 3j-k). These data indicated that inhibition of TAK1 in the CA1 region enhanced pRIPK1 expression and exacerbated surgery-induced neuroinflammation, astrocyte and microglial activation and cognitive impairment in young rats. However, the RIPK1 inhibitor Nec-1 reversed these alterations.

### TAK1 Overexpression in the Hippocampal CA1 Region of Old Rats with POCD

**Fig. 4:**
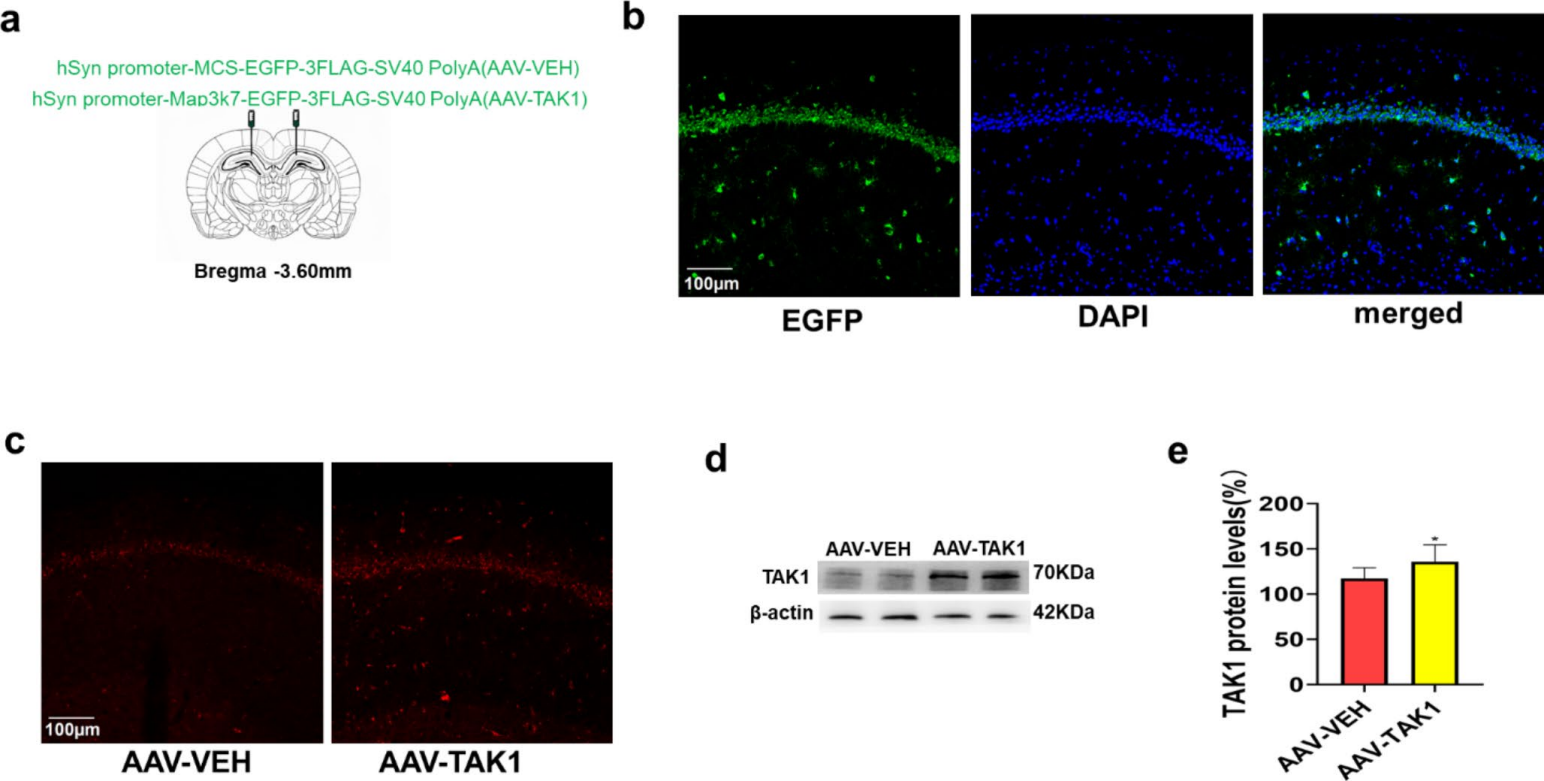
TAK1 overexpression in the hippocampal CA1 region of old rats with POCD. (**a**) Schematic diagram of bilateral virus injections. (**b**) EGFP fluorescence showing the expression of AAV in the CA1 region. (**c**) Analysis of the expression of TAK1 (red) in the CA1 region by immunofluorescence 3 weeks after virus injection. (**d-e**) Representative western blots and analysis of TAK1 expression in the hippocampus. ^*^P < 0.05 vs. the AAV-VEH group. The scale bars indicate 100 μm

To demonstrate the role of TAK1 in surgery-induced POCD development in old rats, as well as the underlying mechanism, the AAV-TAK1/VEH vector was microinjected into the CA1 region of the hippocampus 21 days before surgery (Fig. 4a). Analysis of EGFP fluorescence showed that the AAV-TAK1 vector successfully transduced the CA1 region (Fig. 4b), and the fluorescence intensity of TAK1 was higher in the AAV-TAK1 group than in the AAV-VEH group (Fig. 4c). We also found that the TAK1 expression level was significantly increased in the AAV-TAK1 group compared with the control group (Fig. 4d-e), indicating that overexpression of TAK1 rescued the decrease in TAK1 expression in old rats.

### TAK1 Overexpression Attenuated RIPK1 Activation, Neuroinflammation and Cognitive Dysfunction in Old Rats After Surgery

**Fig. 5:**
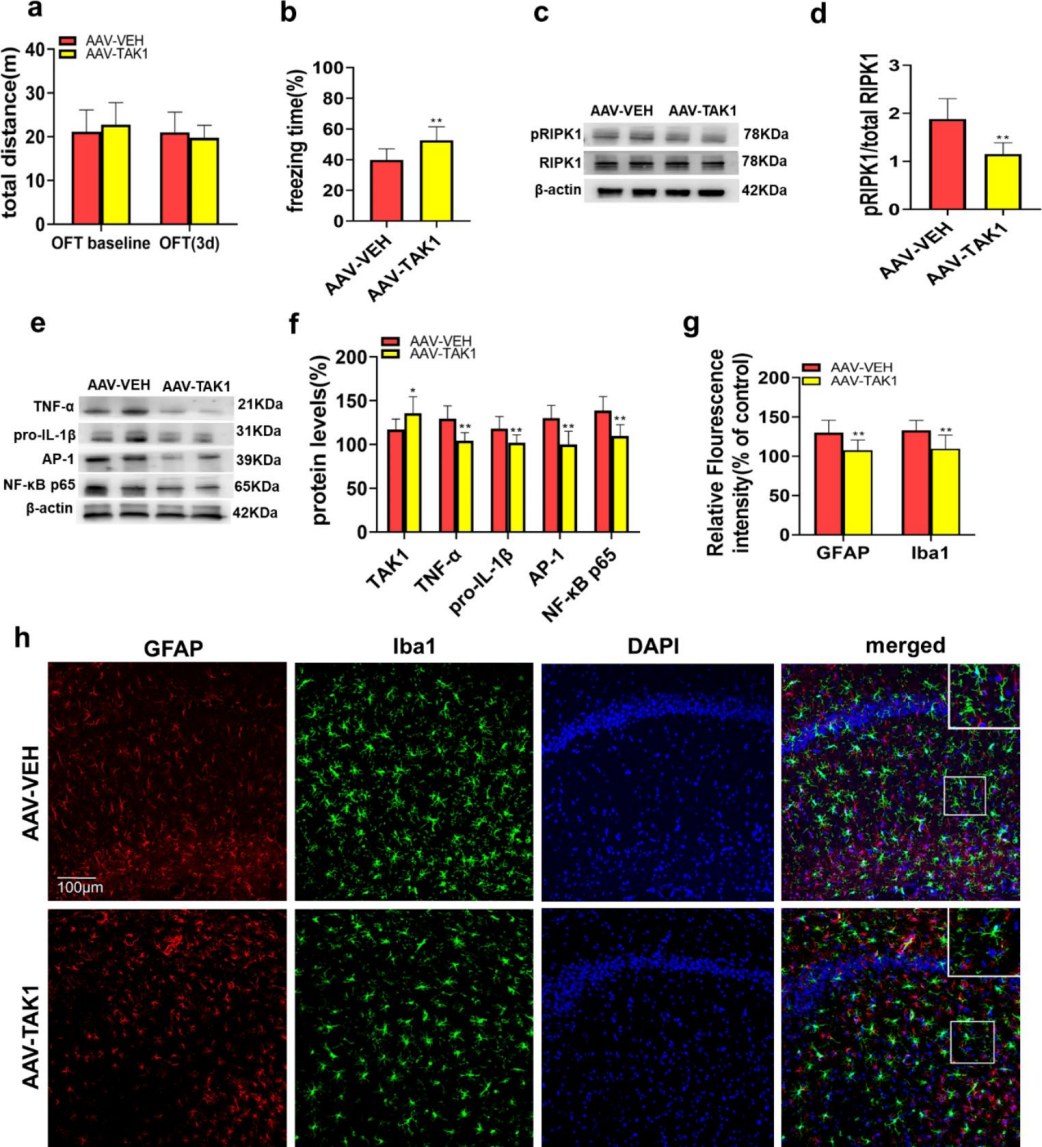
TAK1 overexpression attenuated RIPK1 activation, neuroinflammation and cognitive dysfunction in old rats after surgery. **(a)** Comparison of the total distance travelled by the rats in the OFT at different time points. (**b**) The percentage of freezing time at 72 h after surgery. (**c-d**) Representative western blots and analysis of pRIPK1 and RIPK1 expression in the hippocampus. (**e-f**) Representative western blots and analysis of the TNF-α, pro-IL-1β, AP-1 and NF-κB p65 levels in the hippocampus 72 h after surgery. (**g**) Quantification of GFAP and Iba1 fluorescence in the hippocampal CA1 region 72 h after surgery. (**h**) Representative images of GFAP and Iba1 fluorescence in the hippocampal CA1 region 72 h after surgery. The data are presented as the mean ± SD (n = 10). ^*^P < 0.05, ^**^P < 0.01 vs. the AAV-VEH group. The scale bars indicate 100 μm.

We next aimed to further verify the relationship between TAK1 and RIPK1 and demonstrate the role and mechanism of TAK1 in surgery-induced POCD development in old rats. The OFT was carried out to test locomotor ability in rats. The results showed that virus injection did not affect the motor function of the rats (Fig. 5a). CFC tests were conducted to evaluate cognitive function, and the freezing time in the AAV-TAK1 group was significantly increased compared with that in the control group 72 h postoperatively (Fig. 5b). Then, we measured the levels of proinflammatory cytokines and glial activation in the AAV-VEH and AAV-TAK1 groups by both immunofluorescence and western blotting. Notably, compared with that in the control group, the level of pRIPK1 was significantly decreased in the AAV-TAK1 group (Fig. 5c-d). Next, the TNF-α, pro-IL-1β, AP-1 and NF-κB p65 levels were measured in the two groups. Compared with those in the AAV-VEH group, the levels of TNF-α, pro-IL-1β, AP-1 and NF-κB p65 were decreased in the AAV-TAK1 group (Fig. 5e-f). Similarly, the AAV-TAK1 group had significantly reduced fluorescence intensity of GFAP and Iba1 compared to those of the AAV- VEH group (Fig. 5g-h).

## Discussion

In the present study, we found that old rats had cognitive impairment and marked hippocampal inflammation after surgery, but the surgery did not have this effect in young rats. TAK1 expression levels were lower in old rats that did not receive surgery than in young rats that did not receive surgery and were not altered by surgery. We further found that the TAK1 inhibitor takinib triggered RIPK1 upregulation, astrocyte and microglial activation, hippocampal neuroinflammation and cognitive impairment after surgery, but these changes were reversed by the RIPK1 inhibitor Nec-1 in young rats. Importantly, genetic TAK1 overexpression attenuated the cognitive impairment and neuroinflammation induced by surgery in old rats.

Abundant evidence indicates that individuals who receive anaesthesia and undergo surgery have a high risk of cognitive decline, and this risk increases with age[25, 26]. In our research, we also found that old rats were more susceptible to surgery-induced POCD than young rats. Ageing can impair microglial function and increase susceptibility to proinflammatory activation, thereby promoting ageing-related neurodegeneration[27]. Microglia undergoing cellular senescence exhibit overactivation of the transcription factor NF-κB, which leads to the release of proinflammatory cytokines, such as TNF-α, IL-1β, and IL-6[28]. These proinflammatory cytokines, together with phenotypic changes in astrocytes, oligodendrocytes, neurons, and peripheral immune cells, orchestrate neuroinflammation[29]. In the present study, astrocyte and microglial activation and increased levels of TNF-α, pro-IL-1β, AP-1 and NF-κB p65 were observed in the CA1 region of old rats that developed POCD after surgery. These findings are consistent with our previous studies[30–33]. These results suggest that neuroinflammation after surgery is likely one of the key mechanisms underlying the development of POCD and that overactivation of microglia plays an important role in age-related hippocampal neuroinflammation.

The role of RIPK1 in neurodegenerative diseases has recently been reported [12, 13]. RIPK1 mediates the nuclear transcription factor kappa B (NF-κB)-dependent inflammatory response, caspase-8-dependent apoptosis and mixed-lineage kinase domain-like protein (MLKL)-dependent necroptosis[34, 35]. It has been shown that anaesthesia and surgery induce microglial activation, leading to the synthesis and release of inflammatory cytokines and cognitive decline, and that inhibiting the kinase activity of RIPK1 is effective in attenuating microglia and alleviating cognitive decline[15, 36]. In the current study, we demonstrated that surgery induced RIPK1 activation and neuroinflammation in old rats. This suggests that RIPK1 activation may be involved in the abnormal regulation of central inflammation in individuals with POCD. On the other hand, preoperative injection of Nec-1 into the CA1 region attenuated microglial and astrocyte activation and hippocampal neuroinflammation. This is very likely to be due to its local cellular effect because Nec-1 can easily enter the brain by crossing the compromised blood–brain barrier[14]. One characteristic of ageing is an increase in the permeability of the blood–brain barrier to immune cells and peripheral molecules[37]. These results suggest that RIPK1 may be a target for the development of approaches to prevent and treat POCD in elderly individuals.

TAK1 is an endogenous inhibitor of RIPK1, and an age-dependent reduction in TAK1 expression might be a key factor that contributes to neurodegenerative disease models, such as frontotemporal dementia and amyotrophic lateral sclerosis [18]. As expected, our current study showed that TAK1 expression was decreased in the hippocampus of old rats. We also found that old rats were more susceptible to surgery-induced POCD than young rats. This suggests that an age-dependent reduction in TAK1 expression might be one of the reasons why elderly patients are more likely to develop POCD than young patients after surgery. It has been reported that the dysregulation of RIPK1 suppression might be important in promoting neuroinflammation in the central nervous system[36]. Moreover, TAK1 inactivation (or deficiency) promoted cell death and inflammation[38] *via* RIPK1 upregulation[39]. RIPK1 is suppressed by inhibitory phosphorylation, which is directly mediated by TAK1 and by kinases that are activated by TAK1, including MK2 and IkB kinase (IKKs)[39–42]. Cells that are deficient in the TAK1-mediated suppression of RIPK1 kinase directly promote RIPK1-dependent apoptosis upon stimulation by TNF-a [39, 43, 44]. In our study, the TAK1 inhibitor takinib was injected into the bilateral cerebral ventricle of young rats before surgery; takinib exacerbated surgery-induced pRIPK1 expression, astrocyte and microglia activation, neuroinflammation and cognitive dysfunction, and these abnormities were reversed by the RIPK1 inhibitor Nec-1. Notably, the preoperative injection of AAV-TAK1 into the CA1 region in old rats attenuated cognitive impairment and hippocampal neuroinflammation. Thus, our study indicates that ageing may facilitate RIPK1 activation by decreasing TAK1 expression and subsequently promote POCD development in old rats.

There are some limitations to our study. First, it is well known that the incidence of postoperative cognitive dysfunction is highest within 3 days after surgery [45, 46]. Hence, we used the CFC test to measure early postoperative cognitive function to simulate the clinical situation. Thus, the long-term cognitive changes, particularly those in young animals, are unknown. Second, it is well documented that necroptosis is mediated through the activation of RIPK1 and the subsequent activation of RIPK3, which in turn phosphorylates mixed-lineage kinase domain-like protein, leading to severe inflammation[47–49]. Whether surgical trauma can directly cause these changes remains unknown and warrants further study.

## Conclusions

In summary, the current study demonstrated that the age-dependent reduction in TAK1 expression promotes RIPK1 overactivation, resulting in astrocyte and microglia activation, hippocampal neuroinflammation and POCD in old rats. Hence, the TAK1/RIPK1 signalling pathway may be a potential preventive and therapeutic target for the development of strategies to manage POCD in elderly patients.

## Data Availability

All data generated or analysed during this study are included in this article.
